# Position Effect Variegation and Viability Are Both Sensitive to Dosage of Constitutive Heterochromatin in *Drosophila*

**DOI:** 10.1534/g3.114.013045

**Published:** 2014-07-21

**Authors:** Maria Berloco, Gioacchino Palumbo, Lucia Piacentini, Sergio Pimpinelli, Laura Fanti

**Affiliations:** * Dipartimento di Biologia, Università degli studi di Bari “Aldo Moro,” 70125 Bari, Italy; † Istituto Pasteur, Fondazione Cenci Bolognetti and Dipartimento di Biologia e Biotecnologie “Charles Darwin,” Sapienza Università di Roma, 00185 Roma, Italy

**Keywords:** heterochromatin, PEV, viability, *Drosophila*

## Abstract

The dosage effect of Y-chromosome heterochromatin on suppression of position effect variegation (PEV) has long been well-known in *Drosophila*. The phenotypic effects of increasing the overall dosage of Y heterochromatin have also been demonstrated; hyperploidy of the Y chromosome produces male sterility and many somatic defects including variegation and abnormal legs and wings. This work addresses whether the suppression of position effect variegation (PEV) is a general feature of the heterochromatin (independent of the chromosome of origin) and whether a hyperdosage of heterochromatin can affect viability. The results show that the suppression of PEV is a general feature of any type of constitutive heterochromatin and that the intensity of suppression depends on its amount instead of some mappable factor on it. We also describe a clear dosage effect of Y heterochromatin on the viability of otherwise wild-type embryos and the modification of that effect by a specific gene mutation. Together, our results indicate that the correct balance between heterochromatin and euchromatin is essential for the normal genome expression and that this balance is genetically controlled.

Position effect variegation (PEV) is a well-known case of cis inactivation of a wild-type euchromatic gene when relocated in, or very close to, the heterochromatin. PEV was first described by Muller (1930) in *Drosophila melanogaster*. One of the best examples of PEV is seen when the *white* gene, normally located near the telomere of the *X* chromosome, is transferred by chromosome rearrangement to a new position in the heterochromatin. There, *white* undergoes a *cis*-heterochromatic inactivation during development only in a proportion of the cells of the eyes, giving a mosaic phenotype of mutant and wild-type areas (Spofford 1976). This inactivation of the variegating gene is accompanied by chromatin changes cytologically visible in polytene chromosomes; the *white* region loses its normal morphology, appearing “heterochromatinized” (Shultz and Casperson 1939; Prokofyeva-Belgovskaya 1939; Hartmann-Goldstein 1967; Kornher and Kauffman 1986). A peculiar case of PEV, also observed in *Drosophila*, takes into account the chromosome rearrangements involving the *brown* locus and the pericentromeric heterochromatin. In these cases the variegating *brown* alleles result, consistently dominant over wild-type, thus suggesting a *cis* and *trans* effect with respect to the heterochromatic junction (Muller 1930; Glass 1933; Martin-Morris and Henikoff 1995). It has been shown that the “*trans*-inactivation” is associated with reduced mRNA accumulation of the wild-type gene and requires the pairing between alleles (Henikoff and Dreesen 1989).

In *D. melanogaster*, genetic, physical, and chemical factors that can modify both the *cis* and the *trans* effects on PEV are known (Spofford 1976). The classic suppressor of PEV is the entirely heterochromatic *Y* chromosome. Studies performed on three different genes undergoing PEV have shown that the intensity of suppression is related to the amount of *Y* heterochromatin present in the genome and does not depend on any mappable factor (Dimitri and Pisano 1989). These results are consistent with the hypothesis that the *Y* chromosome competes for free histone and/or nonhistone proteins responsible for the heterochromatinization process diluting these proteins at the variegating sites (Zuckerkandl 1974). Many genetic dominant suppressors of PEV have been isolated (Grigliatti 1991; Reuter and Spierer 1992). These modifiers show dosage effects on PEV in that one dose suppresses and three doses enhance PEV, suggesting a limited production of their proteins (Locke *et al.* 1988). Intriguingly, it has been shown that some suppressors of PEV are recessive lethals, and their lethality depends on their interaction with the *Y* chromosome. For example, a dominant mutation, *Su(var)2-1^01^*, that suppresses position effect variegation (Reuter *et al.* 1982b) displays a lethal interaction with the *Y* chromosome (Reuter *et al.* 1982a): *X/Y* males homozygous for *Su(var)2-1^01^* do not survive, while *X/0* males homozygous for the mutation are viable. Because *Su(var)2-1* induces a significant hyperacetylation of histone H4 (Dorn *et al.* 1986), this lethal interaction has been interpreted as a hyperactivation of the chromatin, producing a strong genetic imbalance due to an accumulation of hyperacetylated histones induced by the suppressor and the titration of heterochromatic proteins by the *Y* chromosome. All these data strongly suggest that heterochromatic proteins are produced in limited amounts, and they raise an important question: if the amount of heterochromatic proteins is critical for the correct functionality of the genome, should hyper-amounts of heterochromatin *per se* affect viability? The phenotypic effects of *Y* heterochromatin dosage, even in wild-type flies, have long been well-known. Cooper (1955) showed that hyperploidy of the *Y* chromosome produces male sterility and many somatic defects including variegation and abnormal legs and wings. More recent data have suggested that quantitative *Y* chromosome polymorphism could be associated with phenotypic variation in both autosomic and *X*-linked gene expression, a phenomenon known as *Y*-linked regulatory variation (YRV) (Francisco and Lemos 2014). We stress that these data are intriguing because in *Drosophila*, the *Y* chromosome is essential only for fertility and it is completely dispensable for viability.

We tested the dosage effects of *X* chromosome and autosomal heterochromatin on PEV and the dosage effects of *Y* heterochromatin on viability. The results show that PEV suppression is a general feature of any type of constitutive heterochromatin, and that the intensity of suppression is related to its amount instead of some mappable heterochromatic factor. Likewise, the lethal interaction of the *Y* with *Su(var)2-1^01^* depends on the overall amount of *Y* heterochromatin and not on a specific site. Importantly, we also discovered a clear dosage effect of *Y* heterochromatin on the viability of otherwise wild-type embryos. All these results indicate that the dosage balance between heterochromatin and euchromatin is essential for viability and that it is genetically controlled.

## Materials and Methods

For a description of chromosome rearrangements and genetic markers, see FlyBase (http://flybase.bio.indiana.edu) and Lindsley and Grell (1968). The majority of free duplications that were generated by same chromosomal inversions share a small euchromatic distal segment. Also, the other free duplications show a cytologically small distal euchromatic segment.

### Culture conditions

Flies were maintained on a standard *Drosophila* medium containing cornmeal, yeast, sucrose, and agar with Nipagin added as a mold inhibitor instead of propionic acid (because the latter can suppress position effect variegation). All cultures were grown at 24°.

### Eye pigment measurement

Heads were collected 3 d after eclosion of the flies by freezing the adults in Eppendorf tubes and vortexing for a few seconds. The red pigment was extracted according to Ephrussi and Herold (1944). Levels were measured using a spectrophotometric assay at 480 nm.

### Mitotic chromosome preparation

Brains were dissected from third instar larvae and mitotic chromosomes were prepared according to Pimpinelli *et al.* (2010).

## Results and Discussion

**Figure 1:**
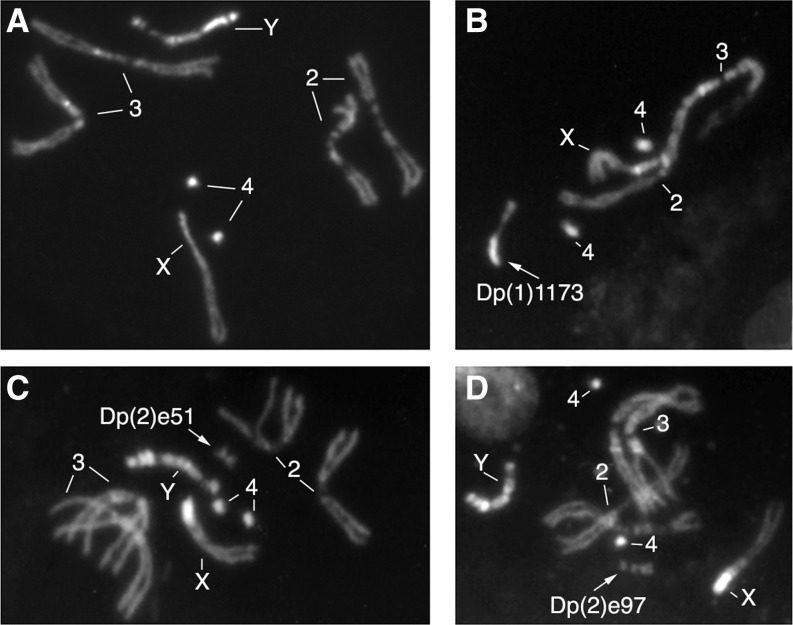
DAPI-stained mitotic chromosomes from *Drosophila* larval neuroblasts. (A) Wild-type karyotype. (B) Karyotype showing, by arrow, the heterochromatic free duplication of the *X* chromosome *Dp(1)1173*. (C, D) Karyotypes showing, by arrows, two different heterochromatic free duplications of a second chromosome: *Dp(2)e51* and *Dp(2)e97*, respectively. The numbers indicate the different autosome pairs, and letters *X* and *Y*, respectively, indicate the *X* and *Y* sex chromosomes.

To assay the effects of autosomal and *X* chromosome heterochromatin on PEV, several different sizes of heterochromatic free duplications derived from an *X* chromosome or a second chromosome (see Figure 1 for examples) were tested on three chromosome rearrangements causing gene variegation. Two of them are inversions of the *X* chromosome: *In(1)l v231*, which carries the variegating lethal *l(1)v231*, and *In(1)w^m4^*, which shows PEV of the wild-type *white* gene. The third rearrangement is *In(2)bw^Vde2^*, an inversion of the second chromosome that carries a variegating allele of the *brown^+^* gene. For all the inversions, the proximal breakpoints are located within the heterochromatin.

### The suppression effect of X heterochromatin on position effect variegation

**Figure 2:**
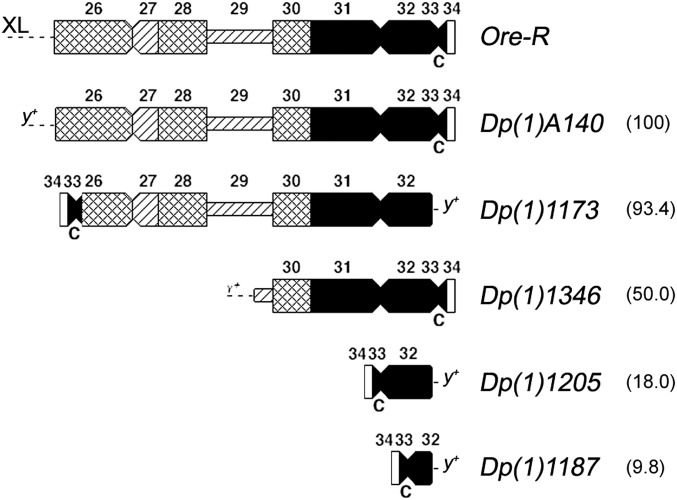
Diagrammatic representation of the DAPI staining pattern of different heterochromatic free duplications of the *X* chromosome. The first diagram above represents the Dapi banding pattern of *X* heterochromatin from the wild-type *Ore-R* strain. The diagrams below show the portions of heterochromatin present in the different free duplications. C indicates the position of the centromere. Region 29 represents the nucleolar organizer. Filled segments indicate bright fluorescence, cross-hatched segments indicate moderate fluorescence, hatched segments indicate dull fluorescence, and open segments indicate no fluorescence. Euchromatin is depicted as a broken line. Note that the free duplications *1173*, *1205*, and *1187* have the centromere positioned on the opposite side with respect to the fluorescence pattern of the heterochromatin in wild-type *X* chromosome. This is because the free duplications were obtained from inversion *In(1)sc^8^*, in which the euchromatic breakpoint is proximal to the *yellow* locus and the heterochromatic breakpoint is close to the centromere. The free duplications derived from *In(1)sc^8^* share the same small distal euchromatic segment. The other free duplication was obtained from a wild-type *X* chromosome and shares a cytologically small euchromatic segment. Numbers inside the brackets indicate the size of the free duplications expressed as a percentage of the wild-type pericentromeric heterochromatin. Note that the duplication *A140* has the whole wild-type heterochromatin.

To test possible effects of *X* heterochromatin on PEV, we used a series of *X* heterochromatic free duplications whose diagrammatic representation is shown in Figure 2. These free duplications were created by Krivshenko and Cooper from the *In(1)sc^8^* and from a wild-type *X* chromosome and described in Lindsley and Grell (1968). The size of these heterochromatic duplications are also reported in Parry and Sandler (1974) and ranges from the shortest *Dp(1)1187* to the longest *Dp(1)A140*, which carries all of the *X* heterochromatin similar to the wild-type *OR-R*.

To test the effects on the variegating lethality caused by *l(1)v231*, we crossed heterozygous females carrying the *l(1)v231* chromosome and a normal *X* chromosome marked with the recessive mutations *yellow (y)*, *white (w)*, and *forked (f)*, with males carrying the attached-*XY* compound chromosome *Y^s^X.Y^L^*, *In(1)EN yB*, and one of the different free duplications of the *X* heterochromatin. This cross produces two types of male progeny, one with *l(1)v231y* and the other with *ywf* ; both carry the same *X* heterochromatic free duplication. The relative viability of the *l(1)v231* chromosome was measured as the ratio of the *l(1)v231y/Dp(1)y^+^* males to their *ywf/Dp(1)y^+^* brothers. Table 1 shows the relative viability of *l(1)v231* males carrying *X* heterochromatic free duplications of different sizes. It is apparent that the viability of the *l(1)v231* males depends on the amount of *X* heterochromatin. Males carrying the smallest free duplication are only 6% as viable as their *ywf* brothers. The viability increases with the amount of heterochromatin to a maximum of 66% viability for the biggest duplication. It is obvious that *X* heterochromatin can suppress the lethality of *l(1)v231* and that the suppression is dosage-dependent. However, the difference in suppression effect between *Dp(1)1173* and *Dp(1)A140* seems to be an exception. In this case, the small size difference (only 7%) corresponds to a remarkable difference in suppression effect (from 46.6% to 66.2%, respectively). Because the euchromatic breakpoint in the *Dp(1)A140* fragment is not precisely mapped, it could be that some genes at the euchromatin–heterochromatin boundary contribute to a PEV-suppression effect.

**Table 1:** Suppression of the *l(1)v231* lethal phenotype by different amounts of the *X* chromosome heterochromatin

*X* Chromosome Heterochromatic Free Duplications	Male Progeny		χ^2^	*P-value*	r.m.v.%*^a^*
	l(1)v231/Dp(1)*y^+^*^b^*	yw/Dp(1)*y^+^			
*Dp(1) 1187*	53	867	720.21	<0.001	6.1
*Dp(1) 1205*	580	4448	1139.07	<0.001	13.0
*Dp(1) 1346*	255	1776	2975.62	<0.001	14.4
*Dp(1) 1173*	1287	2759	535.54	<0.001	46.6
*Dp(1) A140*	1669	2521	173.25	<0.001	66.2

The results are of crosses of heterozygous *l(1)v231/ywf* females for males carrying the attached-*XY* chromosome, *Y^s^X.Y^L^*, *In(1)EN yB*, and the indicated heterochromatic free duplication of the *X* chromosome.

a The suppression effect is expressed as relative male viability (%) *=* $\frac{l\left(1\right)v\mathrm{231}/Dp\left(1\right)y^{+}\;\text{males}}{ywf/Dp\left(1\right)y^{+}\;\text{males}}\times 100$.

b Dp(1)*y^+^ *= X* chromosome heterochromatic free duplications.

The same *X* heterochromatic free duplications were tested for their effects on *In(1)w^m4^* and *In (2)bw^Vde2^*. In the first case, *ywf/ywf* females carrying either one of the *X* heterochromatic free duplications or a *Y* chromosome were crossed to *In(1)yw^m^/^Bs^Y* males. In the second case, the same females were crossed to *X,y/Y*; *bw^v^/Cy* males. In both the experiments, optical density levels in an eye pigment assay were used to determine the effects of heterochromatin dosage. The percentage of suppression was calculated as difference between the optical density levels of progeny with and without the heterochromatic free duplications. Table 2 and Table 3 show that the *X* heterochromatin is also able to suppress the variegation of the *white* and *brown* genes, and that the intensity of suppression is directly related to heterochromatin dosage.

**Table 2:** Suppression of the *white* mottled phenotype by different amounts of the *X* chromosome heterochromatin

	Female Progeny				
*X* Chromosome Heterochromatic Free Duplications	*ywf/yw^m^*; Dp(1)*y^+^ (E)	*ywf/yw^m^* (C)			
	O.D.	O.D.	ΔO.D.*^a^*	± SE	%*^b^*
*Dp(1) 1187*	0.12506	0.07440	0.05066	0.009	14.7
*Dp(1) 1205*	0.12413	0.06463	0.05950	0.014	17.2
*Dp(1) 1346*	0.14489	0.06748	0.07741	0.012	22.4
*Dp(1) 1173*	0.18019	0.07099	0.10920	0.006	31.6
*Dp(1) A140*	0.28508	0.06484	0.22024	0.01	63.8
*Y*	0.40320	0.05790	0.34530	0.007	100.0

The results are of crosses of *ywf/ywf* females carrying the different *X* heterochromatic free duplications, or a *Y* chromosome, for males *In(1)yw^m^/B^s^Y*.

Optical density (O.D. 480 nm) levels were measured in *ywf/yw^m^/Dp(1)y^+^* (E) and *ywf/yw^m^* (C) female offspring.

The pigment assay was based on samples of 10 heads collected 3 d after eclosion of the flies. For each duplication, 10 samples were analyzed.

a *Δ*O.D. = O.D. (E) - O.D. (C).

b Percent of suppression = O.D. *Dp(1)y^+^*/O.D. of the *Y* chromosome.

**Table 3:** Suppression of the *brown* variegated dominant phenotype by different amounts of the *X* chromosome heterochromatin

	Female Progeny				
*X* Chromosome Heterochromatic Free Duplications	*y/ywf*; bw^v^/+/Dp(2)*y^+^ (E)	*y/ywf*; *bw^v^/+* (C)			
*X* duplication	O.D.	O.D.	ΔO.D.*^a^*	± SE	%*^b^*
*Dp(1) 1187*	0.08315	0.06860	0.01455	0.006	3.2
*Dp(1) 1205*	0.10080	0.07540	0.02540	0.006	5.7
*Dp(1) 1346*	0.13729	0.08750	0.04979	0.008	11.1
*Dp(1) 1173*	0.16360	0.05090	0.11270	0.009	25.1
*Dp(1) A140*	0.28690	0.04530	0.24160	0.010	54.0
*Y*	0.48840	0.03910	0.44930	0.016	100.0

The results are of crosses of *ywf/ywf* females carrying the different *X* heterochromatic free duplications, or a *^y+^Y* chromosome, for males *y/Y*; *bw^v^/Cy*.

Optical density (O.D. 480 nm) levels were measured in ywf/y/Dp(1)*y^+^; *bw^v^/+* (E) and *ywf/y*; *bw^v^/+* (C) female offspring.

The pigment assay was based on samples of 10 heads collected 3 d after eclosion of the flies. For each duplication, 10 samples were analyzed.

a *Δ*O.D. = O.D. (E) - O.D. (C).

b Percent of suppression = O.D. Dp(1)y^+^/O.D. of the *Y* chromosome.

### The suppression effect of autosomal heterochromatin on position effect variegation

**Figure 3:**
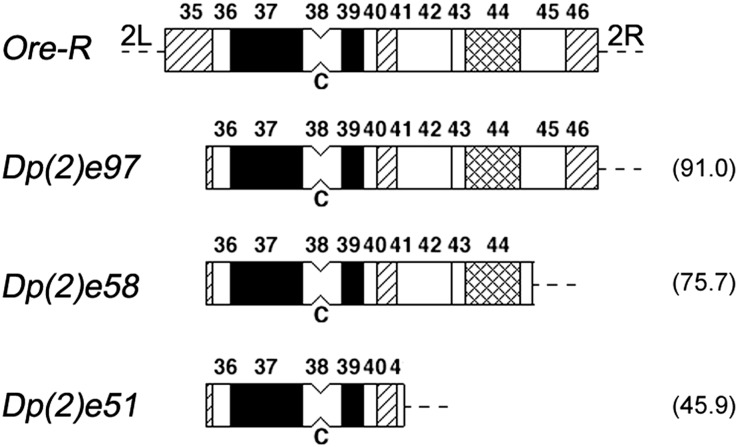
Diagrammatic representation of the DAPI staining pattern of different heterochromatic free duplications of the second chromosome. The first diagram above represents the Dapi banding pattern of the centric heterochromatin of the second chromosome from the wild-type *Ore-R* strain. The diagrams below show the portions of heterochromatin present in the different free duplications. Both the terminal euchromatic regions of 2L and 2R have similar lengths (Brittnacher and Ganetzky 1989). C indicates the position of the centromere. Filled segments indicate bright fluorescence, cross-hatched segments indicate moderate fluorescence, hatched segments indicate dull fluorescence, and open segments indicate no fluorescence. Euchromatin is depicted as a broken line. Numbers inside the brackets indicate the size of the free duplications expressed as a percentage of the wild-type pericentromeric heterochromatin.

To assess the capacity of the autosomal heterochromatin to suppress variegation induced by the same chromosome rearrangements, we used a series of different sizes of heterochromatic free duplications of the second chromosome (Brittnacher and Ganetzky 1989) (Figure 3). Again, we crossed heterozygous females carrying the *l(1)v231* chromosome and a *ywf X* chromosome to males carrying the attached-*XY* compound chromosome *Y^s^X.Y^L^*, *In(1)EN yB*, and one of the free duplications of the second chromosome heterochromatin. Each cross produced two types of male progeny, one carrying the *l(1)v231y* and the other carrying *ywf*, both with the same autosomal heterochromatic free duplication. The relative viability of the *l(1)v231* chromosome was measured as the ratio of the *l(1)v231y/Dp(2)y^+^* males to their *ywf/Dp(2)y^+^* brothers. In Table 4, where the relative viability of *l(1)v231* males carrying autosomal heterochromatic free duplications of different sizes is reported, it appears that the viability of the *l(1)v231* males depends on the amount of autosomal heterochromatin. The viability of males carrying the free duplications compared with their *ywf* brothers ranges from 25.8% for the smallest duplication to 57.9% for the biggest one. Autosomal heterochromatin is also able to suppress the lethality of *l(1)v231* in a dosage-dependent manner.

**Table 4:** Suppression of the *l(1)v231* lethal phenotype by different amounts of the second chromosome heterochromatin

Second Chromosome Heterochromatic Free Duplications	Male Progeny				
	*l(1)v231/0*; Dp(2)*y^+^*^b^*	*ywf/0*; Dp(2)*y^+^	χ^2^	*P-value*	r.m.v.%*^a^*
*Dp(2) e51*	101	391	170.93	< 0.001	25.8
*Dp(2) e58*	221	413	58.15	< 0.001	53.5
*Dp(2) e97*	326	563	63.18	< 0.001	57.9

The results are of crosses of heterozygous *l(1)v231/ywf* females for males carrying the attached-*XY* chromosome, *Y^s^X.Y^L^*, *In(1)EN yB* and the indicated heterochromatic free duplication of the second chromosome.

a The suppression effect is expressed as relative male viability (%) = $\frac{l\left(1\right)v\mathrm{231}/0;\;Dp\left(2\right)y^{+}\;\text{males}}{ywf/0;\;Dp\left(2\right)y^{+}\;\text{males}}\times 100$

b Dp(2)*y^+^ = second chromosome heterochromatic free duplications.

The same autosomal free duplications were tested for their effects on *In(1)w^m4^* and *In (2)bw^Vde2^* chromosome rearrangements. The *ywf/ywf* females carrying the different autosomal heterochromatic free duplications, or a *Y* chromosome, were crossed to *In(1)yw^m^/^Bs^Y* males or to *X,y/Y*; *bw^v^/Cy* males. Once again, we measured the optical density levels in eye pigment assays of progeny carrying or not carrying heterochromatic free duplications. The dosage effect was expressed as the percentage of suppression induced by the different free duplications compared with the *Y* chromosome. Table 5 and Table 6 show a clear dosage effect of autosomal heterochromatin in suppressing the PEV of both *white* and *brown* genes.

**Table 5:** Suppression of the *white* mottled phenotype by different amounts of the second chromosome heterochromatin

	Female Progeny				
Second Chromosome Heterochromatic Free Duplications	*ywf/yw^m^*; Dp(2)*y^+^ (E)	*ywf/yw^m^* (C)			
	O.D.	O.D.	ΔO.D.*^a^*	± SE	%*^b^*
*Dp(2) e51*	0.07399	0.02395	0.05004	0.006	14.5
*Dp(2) e58*	0.15905	0.02805	0.13100	0.01	37.9
*Dp(2) e97*	0.17679	0.02441	0.15238	0.03	44.1
*Y*	0.40320	0.05795	0.34525	0.007	100.0

The results are of crosses of *ywf/ywf* females carrying the different second chromosome heterochromatic free duplications, or *^y+^Y* chromosome, for males *In(1)yw^m^/B^s^Y*. Optical density (O.D. 480 nm) levels were measured in *ywf/yw^m^/Dp(2)y^+^* (E) and *ywf/yw^m^* (C) female offspring.

The pigment assay was based on samples of 10 heads collected 3 d after eclosion of the flies. For each duplication, 10 samples were analyzed.

a *Δ*O.D. = O.D. (E) − O.D. (C).

b Percent of suppression = O.D. *Dp(2)y^+^*/O.D. of the *Y* chromosome.

**Table 6:** Suppression of the *brown* variegated dominant phenotype by different amounts of the second chromosome heterochromatin

	Female Progeny				
Second Chromosome Heterochromatic Free Duplications	*y/ywf*; bw^v^/+/Dp(2)*y^+^ (E)	*y/ywf*; *bw^v^/+* (C)			
	O.D.	O.D.	ΔO.D.*^a^*	± SE	%*^b^*
*Dp(2) e51*	0.18889	0.05778	0.13111	0.022	29.2
*Dp(2) e58*	0.31869	0.05694	0.26175	0.012	58.3
*Dp(2) e97*	0.37689	0.03335	0.34354	0.012	76.5
*Y*	0.48840	0.03912	0.44928	0.016	100.0

The results are of crosses of *ywf/ywf* females carrying the different second chromosome heterochromatic free duplications, or *^y+^Y* chromosome, for males *y/Y*; *bw^v^/Cy*.

Optical density (O.D. 480 nm) levels were measured in *y/ywf*; *bw^v^/+/Dp(2)y^+^* (E) and *y/ywf*; *bw^v^/+* (C) female offspring.

The pigment assay was based on samples of 10 heads collected 3 d after eclosion of the flies. For each duplication, 5 samples were analyzed.

a *Δ*O.D. = O.D. (E) − O.D. (C).

b % = O.D. *Dp(2)y^+^*/O.D. of the *Y* chromosome.

All our data clearly show that heterochromatic free duplications are able to suppress the variegation of all the tested rearrangements and that the intensity of suppression is directly related to the size of the duplications, regardless of the chromosomic origin of the heterochromatin. Because a previous study showed a similar behavior of the entirely heterochromatic *Y* chromosome on PEV (Dimitri and Pisano 1989), this indicates that a dosage effect on PEV is a feature of all constitutive heterochromatin.

### Interaction of different Y chromosome fragments with *Su-var(2)1^01^* mutation

**Figure 4:**
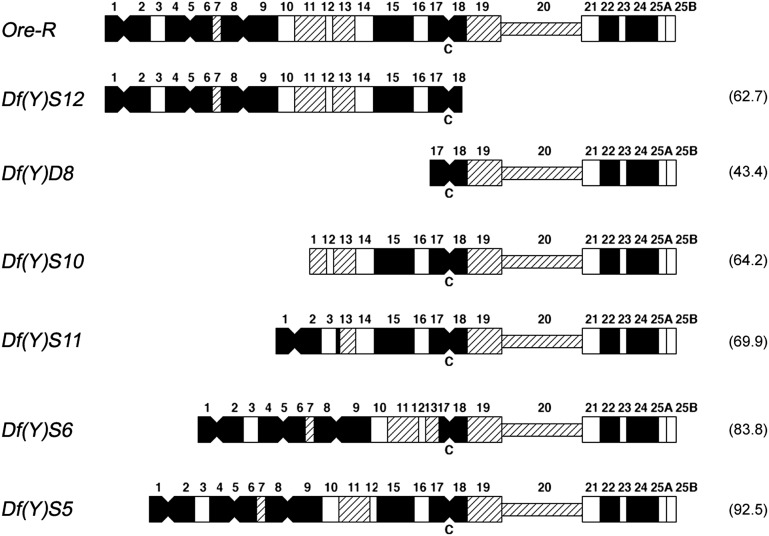
Diagrammatic representation of the DAPI staining pattern of different heterochromatic *Y* chromosome fragments. C indicates the position of the centromere. Region 20 represents the nucleolar organizer. Filled segments indicate bright fluorescence, cross-hatched segments indicate moderate fluorescence, hatched segments indicate dull fluorescence, and open segments indicate no fluorescence. Numbers inside the brackets indicate the size of the free duplications expressed as a percentage of the wild-type *Y* chromosome.

As we mentioned, the dominant PEV suppressor *Su-var(2)1^01^* (Reuter *et al.* 1982b) displays a lethal interaction with the *Y* chromosome. *X/Y* males homozygous for *Su-var(2)1^01^* are completely lethal, whereas homozygous *X/0* males are almost completely viable (Reuter *et al.* 1982a). To test whether the lethal interaction of the *Y* chromosome with *Su(var)2-1^01^* depends on the amount of *Y* heterochromatin, we analyzed the viability of *Su(var)2-1^01^* homozygous males carrying *Y* chromosome fragments of different sizes, as illustrated in Figure 4 (Pimpinelli *et al.* 1985). Table 7 shows the results from crosses of *w^m4^/w^m4^*; *Su(var)2-1^01^/Cy* females with *X-Y*; *Su(var)2-1^01^*, *Sco/+* males, which also carry *Y* chromosome fragments of different sizes, particularly *Df(Y)S6*, *Df(Y)S12*, and *Df(Y)S10* fragments. Fragments *Df(Y)S12 and Df(Y)S10* appear similar in size. However, the length of the nucleolar organizer (region 20 in the diagram of Figure 4) is not representative of its real length because the maps were elaborated from prometaphase chromosomes, where this region is less compact than the rest of heterochromatin. In metaphases, *Df(Y)S12* is significantly longer than *Df(Y)S10*. For each cross, the proportion of the progeny of *Su(var)2-1^01^*, *Sco/Su(var)2-1^01^* homozygous males compared to their *Su(var) 2-1^01^*, *Sco /Cy* heterozygous brothers clearly shows that the lethal interaction is correlated with the size of the *Y* chromosome fragments, thus indicating a quantitative effect of heterochromatin on the lethality induced by the *Su(var)2-1^01^* mutation.

**Table 7:** *Y* chromosome dosage effect on lethal interaction with *Su(var)2-1^01^*

*Y* Chromosome Deficiencies	Male Progeny		χ^2^	*P-value*	r.m.v.%*^a^*
	*Su(var)2-1^01^,Sco/Su(var)2-1^01^*	*Su(var)2-1^01^,Sco/Cy*			
0	647	712	3.11	>0.05	91.00
*Df(Y) S10*	529	723	30.06	<0.001	73.00
*Df(Y) S12*	316	954	320.51	<0.001	33.00
*Df(Y) S6*	137	635	321.25	<0.001	22.00
*Y*	10	184	156.06	<0.001	5.00

The results are from crosses of X-Y/Y*; *Su(var)2-1^0^*, *Sco/+* males for *w^m4^/w^m4^*; *Su(var)2-1^01^/Cy* females.

a The dosage lethal interaction is expressed as a relative male viability (%) = $\frac{Su\left(\mathrm{var}\right)/Su\left(\mathrm{var}\right)/Df\left(Y\right)y^{+}\;\text{males}}{Su\left(\mathrm{var}\right)/+/Df\left(Y\right)y^{+}\;\text{males}}\times 100$.

Y* = *^y+^Y* chromosome deficiencies.

We then analyzed the phenocritical period of the larval lethality. Intriguingly, we found that the majority of the larvae reach the adult stage; the lethality is mainly concentrated at the embryo stage. This suggests a threshold effect of heterochromatin dosage at a restricted and sensitive period during embryo development. The embryos that surmount this stage of sensitivity are able to reach the adult stage.

### The lethal dosage effect of Y chromosome hyperploidy

The phenotypic effects of Y heterochromatin dosage, even in wild-type flies, have long been well-known. Cooper (1955) showed that hyperploidy of the *Y* chromosome produces male sterility and many somatic defects, including variegation and abnormal legs and wings. The somatic dosage effect of the *Y* chromosome is intriguing because this chromosome is essential only for fertility, whereas it is completely dispensable for viability. These data above show a lethal interaction of *Y* heterochromatin with *Su(var)2-1^01^* depending on dosage and suggest that a hyperdosage of heterochromatin could also affect viability in wild-type flies.

We used free duplications of the *Y* chromosome (Figure 4) to determine if altering the dosage of specific *Y* chromosome regions produces phenotypic abnormalities and affects viability. We crossed *ywf/w^+^Y* males, which also carry *Y* chromosome fragments of different sizes to *ywf/ywf/B^s^*Y females, and we analyzed the percentage of male progeny with three *Y* chromosomes, or with two *Y* chromosomes plus another *Y* chromosome fragment. The data reported in Table 8 strongly indicate a quantitative lethal effect of the *Y* heterochromatin. We found lethality among male progeny carrying two *Y* chromosomes plus an additional *Y* fragment, and its strength was related to the fragment size. The only exception seems to be the small reversed effect of *S5* and *S6* with respect to their length. At present, we do not have any plausible explanation. However, we think that this result is not so relevant to affect the general conclusions that lethality is related to the increase of *Y* chromosome dosage. We also observed several phenotypic abnormalities in the surviving progeny with a high dosage of *Y* heterochromatin, such as those described by Cooper (1955).

**Table 8:** *Y* chromosome dosage effect on viability

*Y* Chromosome Deficiencies	ywf/^w+^Y/^Bs^Y/ ^y+^Y* Male Progeny	Total Progeny	Ratio %	± SE
*Df(Y) D8*	66	2650	2.49	0.003
*Df(Y) S10*	67	3651	1.84	0.002
*Df(Y) S11*	52	3724	1.40	0.002
*Df(Y) S6*	18	2993	0.60	0.001
*Df(Y) S5*	18	2776	0.65	0.001
*Y*	6	3267	0.18	0.001

The results are from crosses of ywf/^w+^Y/^y+^Y* males for *ywf/ywf/^Bs^Y* females; Y* = *^y+^Y* chromosome deficiencies.

The dosage effect on viability is expressed as relative male viability % = $\frac{ywf/{}^{w+}Y/{}^{Bs}Y/{}^{y+}Y^{*}\;\text{males}}{\text{total}\;\text{progeny}}\times 100$.

## Conclusions

Our results clearly show that the pericentromeric constitutive heterochromatin of different chromosomes suppresses PEV and that the intensity of suppression is directly related to dosage rather than to any mappable heterochromatic element. We stress that all the types of PEV that we analyzed were due to the relocation of the variegating genes close to pericentromeric heterochromatin. We cannot exclude different sensitivity of telomeric PEV to the dosage of heterochromatin. A different response of telomeric PEV to *Su(var)* mutations has already been shown (Cryderman *et al.* 1999; Wang *et al.* 2014). This suggests that telomeric PEV could be a peculiar silencing mechanism.

**Figure 5:**
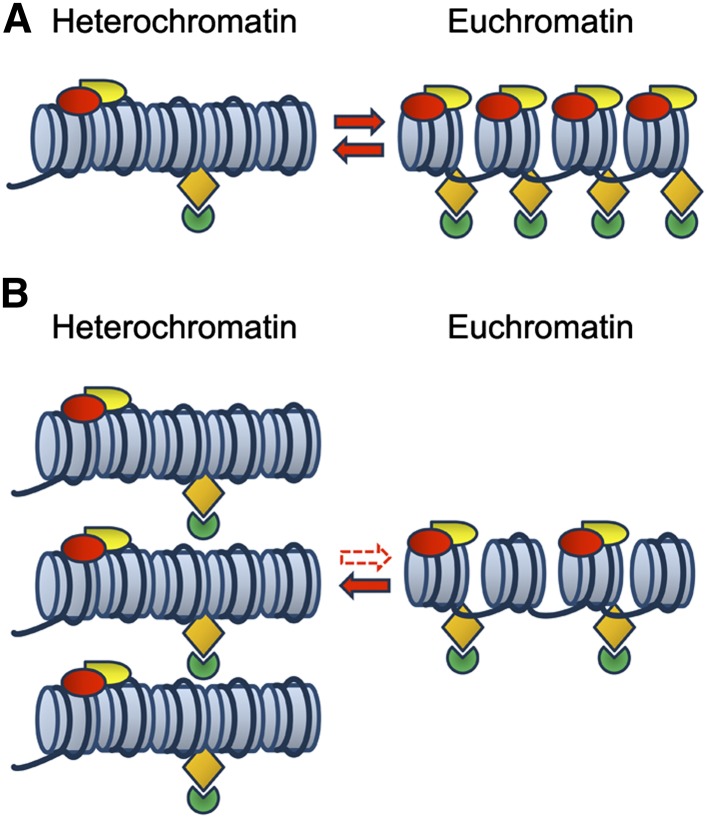
Proposed model suggesting that heterochromatin and euchromatin share several chromatin factors. (A) Wild-type cell where same chromatin factors are present on both the heterochromatic and euchromatic segments in a quantitative equilibrium (opposite oriented arrows). (B) A hyperdosage of heterochromatin would cause a shift of this equilibrium by titration of the shared factors. The consequent loss would induce an impairment of euchromatic functions.

More significantly, the present data show that viability in *D. melanogaster* is also sensitive to the amount of heterochromatin and that this sensitivity can be modified by specific mutations such as *Su(var)2-1^01^*. These data indicate that the correct genome expression depends on the amount of heterochromatin, thus suggesting a functional relationship between heterochromatin and euchromatin. We think that the heterochromatic and the euchromatic domains probably share many structural features involving several chromosomal proteins. Some evidence for a dynamic functional balance between heterochromatin and euchromatin has been already provided by Ebert *et al.* (2004). In this view, the imbalance of genome function produced by a variation in heterochromatin dosage could depend on an alteration in the distribution of chromatin factors between the two domains (Zuckerkandl 1974). This mechanism establishes a functional connection between heterochromatin and euchromatin with heterochromatin regulating euchromatic gene expression by controlling the chromatin structure (Pimpinelli 2000). It is not unreasonable to imagine how a quantitative imbalance of shared proteins between heterochromatin and euchromatin could produce phenotypic effects. A hyper-dosage of heterochromatic DNA may, in fact, accumulate several key regulatory proteins in heterochromatin, thus decreasing their availability for the regulation of normal euchromatic gene expression at various loci (Figure 5). Although other scenarios cannot be ruled out, the demonstration by Fanti *et al.* (2008) that heterochromatin and euchromatin share many chromatin proteins involved in maintaining the expression state of several genes during development seems to support this view.
